# RNA-seq and high-definition mass spectrometry reveal the complex and divergent venoms of two rear-fanged colubrid snakes

**DOI:** 10.1186/1471-2164-15-1061

**Published:** 2014-12-03

**Authors:** James J McGivern, Kenneth P Wray, Mark J Margres, Michelle E Couch, Stephen P Mackessy, Darin R Rokyta

**Affiliations:** Department of Biological Science, Florida State University, Tallahassee, FL 32306-4295 USA; School of Biological Sciences, University of Northern Colorado, Greeley, CO 80639-0017 USA

## Abstract

**Background:**

Largely because of their direct, negative impacts on human health, the venoms of front-fanged snakes of the families Viperidae and Elapidae have been extensively characterized proteomically, transcriptomically, and pharmacologically. However, relatively little is known about the molecular complexity and evolution of the venoms of rear-fanged colubrid snakes, which are, with a few notable exceptions, regarded as harmless to humans. Many of these snakes have venoms with major effects on their preferred prey, and their venoms are probably as critical to their survival as those of front-fanged elapids and viperids.

**Results:**

We sequenced the venom-gland transcriptomes from a specimen of *Hypsiglena* (Desert Night Snake; family Colubridae, subfamily Dipsadinae) and of *Boiga irregularis* (Brown Treesnake; family Colubridae, subfamily Colubrinae) and verified the transcriptomic results proteomically by means of high-definition mass spectrometry. We identified nearly 3,000 nontoxin genes for each species. For *B. irregularis*, we found 108 putative toxin transcripts in 46 clusters with <1% nucleotide divergence, and for *Hypsiglena* we identified 79 toxin sequences that were grouped into 33 clusters. Comparisons of the venoms revealed divergent venom types, with *Hypsiglena* possessing a viper-like venom dominated by metalloproteinases, and *B. irregularis* having a more elapid-like venom, consisting primarily of three-finger toxins.

**Conclusions:**

Despite the difficulty of procuring venom from rear-fanged species, we were able to complete all analyses from a single specimen of each species without pooling venom samples or glands, demonstrating the power of high-definition transcriptomic and proteomic approaches. We found a high level of divergence in the venom types of two colubrids. These two venoms reflected the hemorrhagic/neurotoxic venom dichotomy that broadly characterizes the difference in venom strategies between elapids and viperids.

## Background

Venomous animals have long been studied as a source for drug discovery [1–4] but are increasingly being studied for insight into evolutionary and ecological processes [5–8]. Because of their medically significant bites, some of the best-studied groups of venomous animals are the snakes of the families Elapidae (e.g., cobras, coral snakes, and sea snakes) and Viperidae (e.g., vipers and rattlesnakes). Elapids possess short, fixed front fangs and typically have neurotoxic venoms dominated by three-finger toxins (3FTxs) [9] and type-II phospholipase A toxins (PLA _2_s) [10, 11]. Viperids possess elongate, rotatable front fangs and typically have venoms dominated by enzymatic toxins, such as snake venom metalloproteinases (SVMPs), which cause tissue-damage, bleeding, and necrosis [12, 13]. Relatively little, however, is known about the venoms of rear-fanged snakes (but see Mackessy [14] and Saviola et al. [15]). These venoms are generally less medically relevant because the bites of most rear-fanged species are not lethal to humans, although notable exceptions such as the boomslang (*Dispholidus typus*) exist [16]. In addition, obtaining venom from rear-fanged species in large quantities is generally not possible. The lack of direct human medical consequences and the difficulty in collecting venom from rear-fanged species have left a large gap in our knowledge of snake venoms [14, 17]. Further insight into rear-fanged snake venom may lead not only to the discovery of pharmacologically important proteins, but will also aid in our understanding of the evolution of this complex trait.

The family Colubridae [18] is the largest family of snakes, consisting of seven subfamilies and more than 1,700 species [19]. *Hypsiglena* (subfamily Dipsadinae) is comprised of at least six species of short, stout-bodied, terrestrial, rear-fanged venomous snakes [20]. The genus ranges over a variety of habitats throughout much of western North America, from central Mexico northward throughout the drier regions of the western United States and extreme south-central British Columbia. Members of this genus are largely nocturnal and consume prey as diverse as insect, frogs, and snakes, but more than 70% of their diet consists of lizards and squamate eggs [21]. In contrast, the genus *Boiga* (subfamily Colubrinae) consists of 33 species of long, slender-bodied, arboreal rear-fanged venomous snakes. This nocturnal genus ranges over a variety of habitats across India, southeastern Asia, and northern Australia and is typified by the Brown Treesnake (*Boiga irregularis*). The ecology of this species is relatively well characterized because of its introduction and consequent deleterious effects on the island of Guam [22]. Despite the striking contrasts in geography and life history, the diets of *Hypsiglena* and *B. irregularis* overlap significantly. *Boiga irregularis* consumes mammals, birds, and frogs, but more than 60% of its diet consists of lizards and their eggs [23].

Previous work studying the venoms of rear-fanged species used low-sensitivity methods [24], often requiring the pooling of samples and therefore loss of individual variation [25]. Acquiring venom and gland-tissue in sufficient quantities is challenging for rear-fanged species, but pooling venom from many individuals can confound interpretation of expression and composition. High-throughput transcriptomics [7, 11, 26, 27] and modern proteomic techniques [28–30] can be used to circumvent these issues to characterize venoms in far greater detail than has previously been possible, particularly when both approaches are combined [31]. To better understand the evolution of colubrid snake venoms, we sequenced the venom-gland transcriptomes from *B. irregularis* and a member of an undescribed species of *Hypsiglena* (previously *H. torquata*; hereafter referred to as *Hypsiglena* sp.) from Cochise County, Arizona. These two specimens represent two subfamilies within Colubridae [18]. We used these transcriptomes in conjunction with high-definition mass spectrometry to characterize the venoms of these two species. Because of the high sensitivity of these techniques, we were able to use venom and venom-gland samples collected from a single individual for each species.

## Results and discussion

### Venom-gland transcriptomes

**Figure 1:**
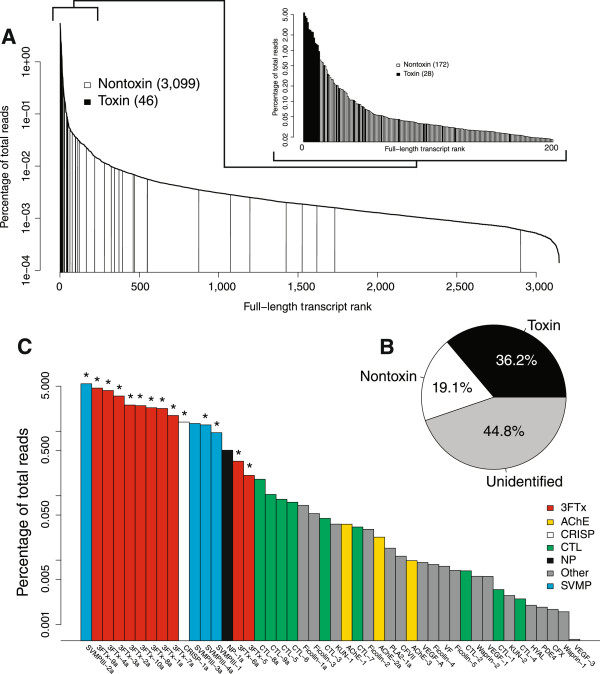
**The venom-gland transcriptome of** ***Boiga irregularis*** **showed high expression and diversity of three-finger toxins.** **(A)** Toxins were overrepresented in the high-abundance transcripts; the 28 most-abundant transcripts encoded toxins. **(B)** Total toxin-gene expression was high and commensurate with values from previously characterized viperids and elapids. **(C)** The toxin transcription consisted primarily of a diverse set of three-finger toxins and a handful of snake venom metalloproteinases. Toxins detected proteomically are indicated with asterisks. Abbreviations: 3FTx–three-finger toxin, AChE–acetylcholinesterase, CF–coagulation factor, CTL–C-type lectin, CRISP–cysteine-rich secretory protein, HYAL–hyaluronidase, KUN-Kunitz-type protease inhibitor, NP–natriuretic peptide, PDE–phosphodiesterase, PLA _2_–Type II Phospholipase A, SVMP–snake venom metalloproteinase, VEGF–vascular endothelial growth factor, VF–venom factor.

We generated 17,103,141 pairs of 151-nucleotide reads that passed the Illumina filter from the venom glands of *B. irregularis*. Of these, 16,324,729 pairs (95.4%) were merged on the basis of their 3’ overlaps. These merged reads had an average length of 142 nucleotides and an average phred quality score of 70. Most reads overlapped over their entire lengths, giving us high confidence in their sequences. The unmerged reads had an average phred quality of 34. Transcriptome assembly and annotation resulted in 3,099 unique nontoxin transcripts with full-length coding sequences and 108 unique putative toxin transcripts (Figure 1 and Table 1). These 108 toxin transcripts were combined into groups with <1% nucleotide divergence in their coding sequences, resulting in 46 distinct clusters. Such clustering facilitates the estimation of transcript abundances and provides a better estimate of the number of toxin sequences.

**Table 1:** **Expression levels of full-length toxin clusters for** ***Boiga irregularis*** **based on 10 million reads mapped to coding sequences**

Rank	Cluster name	Cluster size	CDS length	Median coverage	% Toxin reads	% Total reads
1	SVMPIII-2a	2	1,836	32,071	15.106	5.464
2	3FTx-9a	7	318	116,501	12.923	4.674
3	3FTx-4a	4	318	101,232	11.848	4.286
4	3FTx-3a	11	336	96,068	9.737	3.522
5	3FTx-2a	7	330	69,343	7.058	2.553
6	3FTx-10a	9	318	68,918	6.897	2.495
7	3FTx-8a	6	330	63,436	6.398	2.314
8	3FTx-1a	6	330	60,327	6.242	2.258
9	3FTx-7a	4	330	46,069	4.852	1.755
10	CRISP-1a	4	720	19,816	3.835	1.387
11	SVMPIII-3a	4	1,872	8,563	3.647	1.319
12	SVMPIII-4a	2	1,842	8,043	3.471	1.256
13	SVMPIII-1	1	1,842	6,274	2.630	0.951
14	NP-1a	2	534	9,116	1.400	0.507
15	3FTx-6a	3	255	14,984	0.948	0.343
16	3FTx-5	1	255	8,863	0.572	0.207
17	CTL-8a	2	495	4,283	0.497	0.180
18	CTL-9a	2	483	2,536	0.289	0.105
19	CTL-5	1	498	2,088	0.243	0.088
20	CTL-6	1	591	1,891	0.219	0.079
21	Ficolin-1a	2	996	953	0.196	0.071
22	Ficolin-3	1	996	667	0.146	0.053
23	CTL-3	1	501	1,076	0.123	0.044
24	KUN-1	1	765	623	0.100	0.036
25	AChE-1	1	1,818	268	0.100	0.036
26	CTL-7	1	483	808	0.090	0.033
27	Ficolin-2	1	999	403	0.083	0.030
28	AChE-2a	2	1,650	186	0.063	0.023
29	PLA _2_-1a	2	456	408	0.042	0.015
30	CFVII	1	1,278	117	0.032	0.012
31	AChE-3	1	1,689	54	0.027	0.010
32	VEGF-A	1	579	190	0.025	0.009
33	Ficolin-4	1	1,026	108	0.024	0.009
34	VF	1	4,965	22	0.022	0.008
35	Ficolin-5	1	999	93	0.019	0.007
36	CTL-2	1	531	163	0.019	0.007
37	Waprin-2	1	408	177	0.016	0.006
38	VEGF-1	1	447	153	0.015	0.006
39	CTL-1	1	639	65	0.010	0.003
40	KUN-2	1	1,542	24	0.008	0.003
41	CTL-4	1	507	59	0.007	0.003
42	HYAL	1	1,344	20	0.006	0.002
43	PDE4	1	1,362	18	0.005	0.002
44	CFX	1	1,452	16	0.005	0.002
45	Waprin-1	1	219	82	0.004	0.002
46	VEGF-3	1	627	12	0.002	0.001

*Abbreviations:* 3FTx–three-finger toxin, AChE–acetylcholinesterase, CDS–coding sequence, CF–coagulation factor, CTL–C-type lectin, CRISP–cysteine-rich secretory protein, HYAL–hyaluronidase, KUN-Kunitz-type protease inhibitor, NP–natriuretic peptide, PDE–phosphodiesterase, PLA _2_–Type II Phospholipase A, SVMPIII–snake venom metalloproteinase (P-III), VEGF–vascular endothelial growth factor, VF–venom factor.

**Figure 2:**
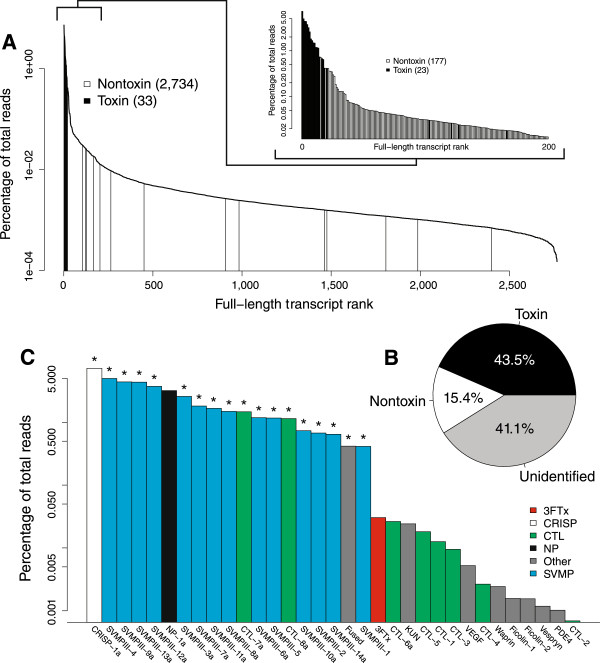
**The venom-gland transcriptome of** ***Hypsiglena*** **sp. showed high expression and diversity of snake venom metalloproteinases.** **(A)** Toxins were overrepresented in the high-abundance transcripts; the 23 most-abundant transcripts encoded toxins. **(B)** Total toxin-gene expression was high and commensurate with values from previously characterized viperids. **(C)** Toxin transcription consisted primarily of a diverse set of snake venom metalloproteinases and the unique Kunitz-Waprin domain fusion protein (i.e., fused toxin). Toxins detected proteomically are indicated with asterisks. Abbreviations: 3FTx–three-finger toxin, CTL–C-type lectin, CRISP–cysteine-rich secretory protein, KUN-Kunitz-type protease inhibitor, PDE–phosphodiesterase, NP–natriuretic peptide, SVMP–snake venom metalloproteinase, VEGF–vascular endothelial growth factor.

For *Hypsiglena* sp., we generated 16,103,579 pairs of 151-nucleotide reads that passed the Illumina filter. Of these, 15,845,565 pairs (98.4%) were merged on the basis of their 3’ overlaps. The average length of the merged reads was 141 nucleotides, and their average phred quality was 72. The unmerged reads had an average quality score of 32. Transcriptome assembly and annotation resulted in 2,734 unique nontoxin transcripts with full-length coding sequences and 79 unique putative toxin transcripts (Figure 2 and Table 2). These 79 toxin transcripts were combined into 33 clusters with <1% nucleotide divergence in their coding sequences.

**Table 2:** **Expression levels of full-length toxin clusters for** ***Hypsiglena*** **sp. based on 10 million reads mapped to coding sequences**

Rank	Cluster name	Cluster size	CDS length	Median Coverage	% Toxin reads	% Total reads
1	CRISP-1a	5	720	92,411	16.684	7.256
2	SVMPIII-4	1	1,836	30,460	11.462	4.985
3	SVMPIII-9a	2	1,833	27,697	10.112	4.398
4	SVMPIII-13a	2	1,836	25,321	10.024	4.360
5	SVMPIII-12a	2	1,830	22,893	8.593	3.737
6	NP-1a	12	474	56,846	7.359	3.201
7	SVMPIII-3a	4	1,860	16,209	5.943	2.585
8	SVMPIII-7a	2	1,857	11,689	4.173	1.815
9	SVMPIII-11a	3	1,845	10,816	3.835	1.668
10	SVMPIII-8a	5	1,845	9,629	3.430	1.492
11	CTL-7a	5	471	32,519	3.382	1.471
12	SVMPIII-6a	3	1,854	7,559	2.727	1.186
13	SVMPIII-5	1	1,833	7,058	2.693	1.171
14	CTL-8a	7	480	25,070	2.633	1.145
15	SVMPIII-10a	4	1,827	4,941	1.687	0.734
16	SVMPIII-2	1	1,827	4,305	1.553	0.675
17	SVMPIII-14a	3	1,839	4,236	1.484	0.645
18	Fused	1	582	8,844	0.960	0.418
19	SVMPIII-1	1	1,857	2,406	0.950	0.413
20	3FTx	1	255	1,357	0.070	0.030
21	CTL-6a	2	480	640	0.060	0.026
22	KUN	1	759	423	0.056	0.024
23	CTL-5	1	477	464	0.042	0.018
24	CTL-1	1	483	304	0.029	0.013
25	CTL-3	1	495	239	0.022	0.010
26	VEGF	1	579	108	0.012	0.005
27	CTL-4	1	483	66	0.006	0.003
28	Waprin	1	405	75	0.006	0.002
29	Ficolin-1	1	1,032	21	0.004	0.002
30	Ficolin-2	1	999	21	0.004	0.002
31	Vespryn	1	558	25	0.003	0.001
32	PDE4	1	1,362	9	0.002	0.001
33	CTL-2	1	531	17	0.002	0.001

*Abbreviations:* 3FTx–three-finger toxin, CDS–coding sequence, CTL–C-type lectin, CRISP–cysteine-rich secretory protein, KUN-Kunitz-type protease inhibitor, PDE–phosphodiesterase, NP–natriuretic peptide, SVMPIII–snake venom metalloproteinase (P-III), VEGF–vascular endothelial growth factor.

As has been described for both elapids [11] and viperids [7, 27], the venom-gland transcriptomes of our two rear-fanged colubrids were extremely biased towards the production of toxin transcripts (Figures 1B and 2B). In *B. irregularis*, approximately 36.2% of the total transcription is accounted for by the coding sequences of our putative toxin-encoding transcripts (Figure 1B), and the 22 most abundant transcripts in the transcriptome encoded putative toxins (Figure 1A). In *Hypsiglena* sp., approximately 43.5% of total transcription was accounted for by the coding sequences of putative toxins (Figure 2B), and the 20 most abundant transcripts in the transcriptome encoded putative toxins (Figure 2A). All of our abundances were based on alignments of reads against only the coding sequences of transcripts.

### Venom proteomes

To verify the transcriptomic results, we conducted proteomic analyses of the venoms of both species using venom from the transcriptome animals. Nanospray LC/MS^E^ analysis of the whole venom of *B. irregularis* identified peptide evidence for 14 of the 45 (31.1%) putative toxin transcript clusters using a database generated from all of the unique transcripts (toxin and nontoxin) identified in the transcriptome. Transcripts identified by means of LC/MS^E^ represented three toxin classes (Table 3), including three distinct clustersof SVMPs (identifying two alleles of cluster 2), 10 unique clusters of 3FTxs, and two allelic variants of the cysteine-rich secretory protein (CRISP) cluster. In addition to distinguishing between transcript clusters within toxin families, our approach was sensitive enough to distinguish between alleles within clusters (Table 3). The SVMPIII-2 cluster consisted of two sequences which differed at three nucleotide sites and three amino acid positions. The frequencies of the variants at these positions were all between 41.4–55.0%, suggesting that these transcripts were two alleles of a single locus. The CRISP-1 cluster consisted of four sequences, and we were able to distinguish CRISP-1a and CRISP-1c from CRISP-1b and CRISP-1d. These two subgroups differed by a single nonsynonymous mutation with the b/d variant at a frequency of 39.7% in the transcriptome. Our whole venom proteomic approach was sensitive enough to detect these minor differences but failed to detect proteins corresponding to the majority of the putative toxin transcripts (Figure 1). Most of the undetected transcripts were in the low-abundance tail of expression levels (Figure 1) and may have fallen below a detection threshold. Alternatively, the low expression levels may indicate that these putative toxins are in fact not toxins and do not contribute to the venom. Two high-abundance putative toxin transcripts for *B. irregularis* were not detected: a natriuretic peptide (NP-1a) and SVMPIII-3a (Figure 1 and Table 3). Detection of NPs is often complicated because this class of toxin undergoes significant post-translational processing during maturation [32]. The failure to detect SVMPIII-3a is more difficult to explain because it is expressed at higher levels than two other SVMPs (Figure 1) that were detected. The sequence has a clear signal peptide but was the most divergent cluster of the four SVMP clusters for *B. irregularis*.

**Table 3:** ***Boiga irregularis*** **LC/MS** ^**E**^ **protein identifications**

Transcript	PLGS score	Peptide	% Seq.	
name	score	matches	coverage	Group
SVMPIII-1	896.04	29	57.84	1
SVMPIII-2b	2,805.59	26	62.77	2
SVMPIII-2a	2,694.91	29	66.50	3
SVMPIII-4a	900.64	29	64.92	3
SVMPIII-4b	890.08	30	64.92	3
CRISP-1b	6,457.39	23	93.67	4
CRISP-1d	6,198.10	21	85.52	4
CRISP-1a	6,248.59	21	87.33	5
CRISP-1c	6,507.89	23	95.48	5
NatA-10	327.40	5	7.92	6
3FTx-1a	4,423.76	10	78.89	7
3FTx-1b	6,549.43	10	78.89	7
3FTx-1c	4,423.76	10	78.89	7
3FTx-1d	6,549.43	10	78.89	7
3FTx-1e	6,549.43	10	78.89	7
3FTx-1f	4,423.76	8	74.44	7
3FTx-3a	8,140.22	6	40.22	8
3FTx-3b	8,140.22	6	40.22	8
3FTx-3c	8,140.22	6	40.22	8
3FTx-3d	2,067.74	4	20.65	8
3FTx-3e	8,140.22	6	40.22	8
3FTx-3f	8,140.22	6	40.22	8
3FTx-3g	8,140.22	6	40.22	8
3FTx-3h	8,140.22	6	40.22	8
3FTx-3i	8,140.22	6	40.22	8
3FTx-3j	6,072.48	2	19.78	8
3FTx-3k	2,067.74	4	20.65	8
3FTx-4a	7,234.41	15	100.00	9
3FTx-4b	6,945.58	13	87.21	9
3FTx-4c	5,137.73	14	94.19	9
3FTx-4d	7,234.41	14	92.94	9
3FTx-5	480.09	2	53.97	10
3FTx-6b	480.09	2	53.97	10
3FTx-6a	1,252.34	3	53.97	11
3FTx-6c	1,252.34	3	54.84	11
3FTx-7a	6,785.76	11	66.67	12
3FTx-7b	6,815.80	12	78.89	12
3FTx-7c	6,785.76	11	66.67	12
3FTx-7d	2,506.53	8	62.22	12
3FTx-8a	3,911.73	9	51.11	13
3FTx-8b	3,911.73	9	51.11	13
3FTx-8c	3,911.73	9	51.11	13
3FTx-8d	3,911.73	9	51.11	13
3FTx-8e	3,911.73	9	51.11	13
3FTx-8f	3,911.73	8	39.33	13
3FTx-9a	9,625.12	14	81.40	14
3FTx-9b	9,625.12	14	81.40	14
3FTx-9c	9,625.12	14	81.40	14
3FTx-9d	9,625.12	14	81.40	14
3FTx-9e	9,625.12	13	69.41	14
3FTx-9f	9,625.12	13	69.41	14
3FTx-9g	9,625.12	14	81.40	14
3FTx-10a	3,753.53	10	75.58	15
3FTx-10b	4,914.06	13	91.86	15
3FTx-10c	3,753.53	10	79.27	15
3FTx-10d	4,914.06	12	84.71	15
3FTx-10e	4,914.06	12	84.71	15
3FTx-10f	3,530.34	10	69.77	15
3FTx-10g	4,914.06	13	91.86	15
3FTx-10h	4,914.06	13	91.86	15
3FTx-10i	4,914.06	13	76.85	15
3FTx-2a	6,549.43	9	55.56	–
3FTx-2b	6,549.43	9	55.56	–
3FTx-2c	6,549.43	9	55.56	–
3FTx-2d	4,423.76	9	55.56	–
3FTx-2e	6,549.43	9	55.56	–
3FTx-2f	5,175.20	6	34.44	–
3FTx-2g	3,049.53	6	34.44	–

Transcripts were grouped on the basis of shared, unique peptides. Identifications without a group designation lacked unique identifying peptides but still had peptide matches. Abbreviations: 3FTx–three-finger toxin, CRISP–cysteine-rich secretory protein, SVMPIII–snake venom metalloproteinase (P-III).

For *Hypsiglena* sp., we identified peptide evidence for 18 of the 33 (54.5%) putative toxin transcript clusters. The identified toxins belonged to four classes (Figure 2 and Table 4), including a previously proteomically unverified fused toxin containing waprin and Kunitz-type protease inhibitor domains. A similar putative toxin was detected in the venom-gland transcriptome of the viperid *Sistrurus catenatus edwardsii*[33, 34]. We proteomically verified the secretion of at least two unique alleles of CRISP, two clusters of C-type lectins (CTLs), and 14 clusters of SVMPs (Figure 2 and Table 4). We also found peptide evidence for hemoglobin subunit *β*1, suggesting that a small amount of blood was mixed with the venom during extraction. The only high-abundance putative toxin transcript for which we failed to detect a corresponding protein in the venom was NP-1, probably for the same reasons we failed to detect the orthologous protein in the venom of *B. irregularis*.

**Table 4:** ***Hypsiglena*** **sp. LC/MS** ^**E**^ **protein identifications**

Transcript	PLGS	Peptide	% Seq.	
name	score	matches	coverage	Group
CRISP-1b	90,678.90	28	97.29	1
CRISP-1d	90,678.90	28	97.29	1
CRISP-1a	83,532.05	29	97.29	2
CRISP-1c	83,532.05	29	97.29	2
CRISP-1e	80,437.63	27	92.56	2
CTL-7e	36,677.55	17	96.24	3
CTL-7d	36,677.55	14	63.91	3
CTL-7b	36,677.55	17	96.24	3
CTL-7a	36,677.55	17	96.24	3
CTL-7c	36,564.01	15	84.96	3
CTL-8g	26,033.11	19	82.22	4
CTL-8f	25,919.58	18	81.62	4
CTL-8e	25,919.58	18	82.22	4
CTL-8d	25,919.58	18	81.62	4
CTL-8c	25,919.58	18	81.62	4
CTL-8b	25,919.58	18	81.62	4
CTL-8a	25,919.58	18	81.62	4
Fused	3,986.49	9	53.45	5
SVMPIII-1	2,196.46	23	41.14	6
SVMPIII-3a	6,757.38	33	44.41	7
SVMPIII-3b	6,757.38	34	44.41	7
SVMPIII-3c	6,754.13	33	44.41	7
SVMPIII-3d	6,754.65	32	44.41	7
SVMPIII-4	22,516.59	42	59.05	8
SVMPIII-5	5,473.71	36	43.56	9
SVMPIII-6a	4,589.49	27	39.20	10
SVMPIII-6b	4,596.38	29	39.20	10
SVMPIII-6c	4,589.49	27	39.20	10
SVMPIII-7a	10,796.01	28	61.87	11
SVMPIII-7b	10,792.75	27	61.87	11
SVMPIII-8a	3,825.85	26	28.45	12
SVMPIII-8b	3,825.85	26	28.45	12
SVMPIII-8c	3,940.07	29	26.94	12
SVMPIII-8d	3,855.83	27	28.45	12
SVMPIII-8e	3,848.95	25	28.45	12
SVMPIII-9a	22,893.75	38	59.32	13
SVMPIII-9b	22,893.75	38	59.32	13
SVMPIII-10a	1,531.99	23	27.04	14
SVMPIII-10b	1,531.99	23	27.04	14
SVMPIII-10c	1,531.99	23	27.04	14
SVMPIII-10d	1,525.10	22	27.04	14
SVMPIII-11a	6,924.50	33	45.29	15
SVMPIII-11b	6,893.95	32	42.91	15
SVMPIII-11c	6,901.40	33	43.60	15
SVMPIII-12a	9,702.24	42	56.54	16
SVMPIII-12b	9,702.24	42	56.54	16
SVMPIII-13a	16,451.10	38	54.15	17
SVMPIII-13b	16,460.75	39	54.15	17
SVMPIII-14a	4,436.73	34	39.86	18
SVMPIII-14b	4,436.73	34	38.56	18
SVMPIII-14c	4,442.71	37	39.86	18
Hemoglobin-b1	1,105.63	6	44.22	19
SVMPIII-2	5,775.72	40	39.80	–

Transcripts were grouped on the basis of shared, unique peptides. Identifications without a group designation lacked unique identifying peptides but still had peptide matches. Abbreviations: CTL–C-type lectin, CRISP–cysteine-rich secretory protein, SVMPIII–snake venom metalloproteinase (P-III).

### The elapid-like venom of *Boiga irregularis*

Combining the transcriptomic and proteomic characterizations of the venom of *B. irregularis*, we found that this long, slender, largely arboreal colubrid has venom redolent of the venoms of its elapid cousins. The most abundant and diverse toxin class for *B. irregularis* was the 3FTxs (Figure 1C and Table 1). Three-finger toxins possess a conserved structure of three loops, which are stabilized by disulfide bridges, extending from a central core [35]. These toxins are often neurotoxic, selectively binding muscarinic [36] and adrenergic [37] receptors. In *B. irregularis*, 3FTxs have been shown to possess toxicity specific to birds and lizards [38]. We identified 58 unique 3FTx sequences that grouped into 10 clusters. These 10 3FTx clusters accounted for 67.5% of the toxin-reads and 24.4% of the total transcription. All ten clusters of 3FTxs were proteomically confirmed and could be divided into three groups on the basis of their lengths and conserved cysteine residues. Of these three groups, the smallest contained clusters 3FTx-5 and 3FTx-6 and was most closely related to 3FTx-Tel1 (Genbank accession: EU029671) previously isolated from the venom of the colubrid *Telescopus dhara* (the Arabian Cat Snake) [39]. These two clusters of sequences from *B. irregularis* differed from 3FTx-Tel1 by 14.2% and 18.6% at the amino-acid level, respectively. This group was characterized by nine conserved cysteine residues and a total length of 63 amino-acids after removal of the predicted signal peptides. The two sequences in this group differed by 4.9% at the amino-acid level. The second largest group consisted of clusters 3FTx-4, 9, and 10. These sequences shared 10 conserved cysteine residues and an amino-acid length of 86. The sequences in this group had pairwise amino-acid differences ranging from 16.9–18.1%. The largest group was composed of clusters 3FTx-1, 2, 3, 7, and 8. These sequences had 10 conserved cysteine residues and lengths ranging from 90–92 amino-acids after removing predicted signal peptides. Interestingly, none of these 3FTx sequences were particularly similar to Irditoxin (Genbank accession: DQ304538), a 3FTx previously isolated from *B. irregularis*[40] with properties consistent with this group. All our 3FTx sequences showed ≥6.7% amino-acid divergence. This lack of a close match to Irditoxin may reflect the different geographic origins of our animal compared to that of Pawlak et al. [40]. The sequences in this group have pairwise amino-acid differences ranging from 7.7–33.9%.

Three-finger toxins are among the most common and diverse venom components in elapids but are only rarely detected in viperids. For the elapid *Micrurus fulvius*, 3FTxs were the second most diverse and highly expressed class of venom genes in the venom-gland transcriptome [11]. Similarly, for the elapids *Ophiophagus hannah* (King Cobra)[8] and *Bungarus flaviceps* (Red-headed Krait) [41], 3FTxs were the most abundant venom transcripts in the venom-gland transcriptomes. In contrast, of the numerous viperid venom-gland transcriptomes that have been characterized [7, 27, 42], few have shown evidence of 3FTxs. The transcriptome of *Sistrurus catenatus edwardsii* showed evidence for 3FTxs, but this evidence consisted of five distinct transcripts at extremely low abundances [33]. A 3FTx was also detected at low levels in the transcriptome of *Protobothrops flavoviridis*[43] and in the venom proteome of *Atropoides nummifer*[44]. Three-finger toxins have been described for colubrids [6, 39, 40, 45], but our results show that colubrid venoms can be as diverse and specialized for 3FTxs as the venoms of elapids.

The highest-abundance individual transcript was a SVMP, and, overall, *B. irregularis* expressed four clusters of SVMP, although only three of these were detected proteomically (Figure 1). The coding sequences from these four clusters accounted for 24.9% of the toxin-reads and 9.0% of the total reads. All of these SVMPs were class P-III [46], possessing both a disintegrin-like domain and a cysteine-rich domain in addition to the metalloproteinase domain [47]. Class P-III snake venom metalloproteinases are capable of rapid hemorrhagic activity by degrading the basement membranes and adhesion proteins and disrupting structural components of the tissues [13, 48]. They are generally associated with the hemorrhagic venoms of viperids [49], but they are also well-represented in the venoms of elapids [50, 51]. For example, the venom-gland transcriptome of the eastern coral snake (*Micrurus fulvius*) had moderate levels of SVMP expression [11].

The only other putative toxin detected proteomically for *B. irregularis* was a single cluster of CRISP, a toxin class identified in the venom of *B. irregularis* in a previous study [38]. Cysteine-rich secretory protein transcripts accounted for 3.8% of the toxin transcription and 1.4% of the total transcription (Figure 1). These toxins have diverse functions. In snake venoms, they have been shown to block cyclic nucleotide-gated and voltage-gated ion channels and inhibition of smooth muscle contraction, potentially disrupting homeostasis [52]. Their full role in envenomation, however, is still unclear [53]. Cysteine-rich secretory proteins are widespread in reptile venoms and well-represented in both viperid and elapid venoms [53].

In addition to the proteomically verified classes of toxin, we transcriptomically identified a number of additional putative toxins that may be important components of the venom of *B. irregularis* (Figure 1). Our failure to detect these proteomically may reflect a limitation of our whole-venom proteomic approach that was made necessary by the low venom yields of both of our species as discussed above. Nonetheless, these remaining toxins should generally be viewed as putative and unconfirmed. Hill and Mackessy [24] detected acetylcholinesterase (AChE) activity in the venom of *B. irregularis*, and we likewise detected three transcript clusters of AChE (Figure 1). At least one of these clusters therefore probably encodes a significant venom component. Acetylcholinesterase activity is generally widespread in elapid venoms [54] but not viperid venoms [43]. These enzymes are capable of rapidly inactivating the neurotransmitter acetylcholine, thereby interfering with neuronal signaling mechanisms. Our single cluster of NP was expressed at high levels (1.4% of the toxin reads) and was probably not detected because of complex post-translational processing these toxins undergo [32]. These peptides are known to have diuretic and vasodilatory function [55]. We detected nine clusters of C-type lectins (CTLs), but altogether these transcripts only account for 1.5% of the toxin reads (0.5% of the total reads). Toxic CTLs possess high sequence homology with the previously discovered carbohydrate recognition domains of non-toxic C-type lectins [56]. Many of these non-enzymatic toxins have been discovered in snake venoms. They are composed of dimers or multimers, shown to bind carbohydrate residues and are implicated in anticoagulant and platelet modulating functions. We detected four clusters of ficolins, which have been found in the transcriptome and venom proteome of the colubrid *Cerberus rynchops* (dog-faced watersnake) [57], but they were expressed at low levels and were not detected proteomically. These putative toxins share sequence homology with the mammalian ficolin, including collagen- and fibrinogen-like domains. The bioactivity of the their non-toxic counterparts suggests that they may possess toxic lectin activity and bind N-Acetylglucosamine [58]. We also transcriptomically detected two Kunitz-type protease inhibitors (KUN), a phospholipase A2 (PLA _2_), coagulation factors (CF) VII and X, three vascular endothelial growth factors (VEGFs), venom factor (VF), two waprins (WAP), one phosphodiesterase (PDE), and hyaluronidase (HYAL). Mackessy and Hill [24] explicitly tested for HYAL activity in the venom of *B. irregularis* and failed to detect it.

Combining our proteomic and transcriptomic results with previous work [24, 38], we can conclude that *B. irregularis* has a distinctly elapid-like, neurotoxic venom. The primary components were a diverse array of 3FTxs. Cysteine-rich secretory protein, NP, and AChE also appeared to be significant components of the venom. Snake venom metalloproteinases were also present but not particularly diverse, a pattern similar to that seen for the elapid *M. fulvius*[11]. Given the evolutionary propinquity of colubrids and elapids, this similarity is not surprising.

### The viperid-like venom of *Hypsiglena*

The venom of *Hypsiglena* sp. was more similar to the hemorrhagic viperid venoms than to the neurotoxic venoms typical of its closer relatives, the elapids. By far the most abundant and diverse class of toxins in the transcriptome was the SVMPs. The 14 clusters of SVMPs accounted for 68.7% of the toxin transcription and 29.9% of the total venom-gland transcription. All 14 clusters of SVMP were verified proteomically (Figure 2). This level of diversity and expression was comparable to SVMPs in viperids such as *Protobothrops flavoviridis*[43] and *Crotalus adamanteus*[27], although *Hypsiglena* sp. only had class P-III SVMPs, whereas three classes of SVMP are known from viperids [46].

The remaining proteomically confirmed toxins include a CRISP, which at 16.7% of the toxin transcription was the most highly expressed putative toxin. Cysteine-rich secretory proteins were also detected proteomically in the venom of *H. torquata texana*[17]. C-type lectins are generally common, diverse, and highly expressed in viperid venoms [59], and we identified eight clusters of CTLs, but only two of these were detected proteomically (Figure 2). Finally, we detected an unusual putative toxin with Kunitz-type protease inhibitor and WAP domains in the transcriptome, which was later confirmed in the venom proteome. This fused toxin was similar to sequences identified in the transcriptomes of the viperids *Sistrurus catenatus edwardsii*[33, 34], *Protobothrops flaviviridis*, and *Ovophis okinavensis*[43]. Although we did not detect the product of the NP transcript proteomically, its high expression level (7.4% of the toxin expression) suggests that this is an important component of the venom.

The remaining toxin classes probably represent minor functional components of the venom. In addition to six of the eight CTLs, we also failed to detect proteomically the single 3FTx cluster, a KUN, a VEGF, a WAP, two ficolins, a vespryn (VESP), and a PDE (Figure 2). Three-finger toxins were the major components of the venom of *B. irreglaris* but were represented by a single cluster accounting for just 0.07% of the toxin reads for *Hypsiglena* sp. Weak proteomic evidence for 3FTxs in the venom of *H. torquata texana* has been described [17], but, if 3FTxs were present in the venom of our specimen of *Hypsiglena* sp., they were obviously very minor components.

The venom of *Hypsiglena* sp. consisted primarily of SVMPs and the nonenzymatic CRISP, NP, and fused toxin. Hill and Mackessy [24] tested for various enzymatic activities in the venom of *H. torquata texana* and were only able to detect proteolytic activity, which is in agreement with our results. With its abundant and diverse SVMPs and CTLs, the venom of *Hypsiglena* sp. showed a distinct similarity to typical viperid venoms, in contrast with the elapid-like venom of *B. irregularis*.

### Selection in colubrid toxins

**Figure 3:**
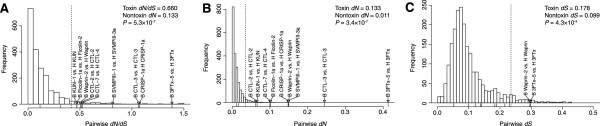
**Pairwise comparisons of evolutionary rates for toxins and nontoxins.** The histograms show the distributions of the **(A)** ratio of pairwise nonsynonymous to synonymous substitution rates (*d* *N*/*d* *S*), the **(B)** pairwise nonsynonymous substitution rates (*dN*), and the **(C)** pairwise synonymous substitution rates (*dS*) for the nontoxins. The vertical dashed lines represent the 95th percentile of the nontoxin values. The values for the toxin are shown as a rug plot, with values above the 95th percentile for the nontoxins indicated by gray triangles. *P*-values were based on Wilcoxon rank sum tests. Abbreviations: 3FTx–three-finger toxin, AChE–acetylcholinesterase, CTL–C-type lectin, CRISP–cysteine-rich secretory protein, KUN-Kunitz-type protease inhibitor, SVMP–snake venom metalloproteinase.

The strongest and most consistent molecular evolutionary pattern in toxin-protein coding sequences of both elapids [11, 60] and viperids [7, 26, 60] has been the presence of diversifying selection in the form of high ratios of nonsynonymous to synonymous substitutions. Sustained coevolution between snakes and their predators or prey could provide the requisite selection to drive this pattern, making this evolutionary signal a potentially powerful indicator of toxic function [61–63]. To determine whether such patterns also characterized the putative toxins identified for *B. irregularis* and *Hypsiglena* sp., we conducted several selection analyses. The first analysis mirrored that of Rokyta et al. [7] of generating null distributions of pairwise evolutionary rates on the basis of nontoxic orthologs from the venom-gland transcriptomes. From our 3,099 (*B. irregularis*) and 2,734 (*Hypsiglena* sp.) nontoxins, we identified 2,069 orthologs by means of reciprocal blast. Similarly, we identified 11 putatively orthologous toxin pairs from these two species. The toxin pairs showed significantly higher pairwise synonymous (*dS*; *P*=4.3×10^−4^; Figure 3C) and nonsynonymous (*dN*; *P*=3.4×10^−7^; Figure 3B) divergence. The toxins also showed a significantly higher ratio of nonsynonymous to synonymous substitution rates (*dN*/*dS*; *P*=5.3×10^−7^; Figure 3A). We used the nontoxin distributions to generate 95% thresholds for these rates for nontoxin sequences and found that nine of the 11 pairs of toxins exceeded these thresholds for *dN* and *dN*/*dS*, but only two exceeded the *dS* threshold (Figure 3). If the toxin and nontoxin distributions were the same, we would expect to see less than one toxin pair exceed the threshold determined by the nontoxin pairs. Our putative toxins therefore appeared to be evolving at higher rates than the nontoxins, particularly in terms of nonsynonymous substitutions. Only three pairs of toxins (a CTL, a 3FTx, and a CRISP pair) showed *dN*/*dS*>1, a conservative [64, 65] indicator of positive or diversifying selection.

For the larger toxin-gene families identified for *B. irregularis* and *Hypsiglena* sp. (SVMPs, 3FTxs, CTLs, and ficolins), we used phylogenetic methods to determine whether positive selection was acting on sites within these genes. The SVMPs were the largest class with four and 14 representatives from *Hypsiglena* sp. and *B. irregularis*, respectively (Figures 1 and 2; Tables 1 and 2). These class P-III SVMPs had two functional domains in addition to the metalloproteinase domain: a disintegrin-like domain and a cysteine-rich domain. We analyzed these three domains separately for evidence of selection. For the CTLs, we were only able to include 12 of the total 17 sequences because of excessive sequence divergence. Using both the M1/M2 and M7/M8 model comparisons in codeml [66, 67], we identified evidence of a class of sites undergoing positive selection in all six alignments considered (Tables 5 and 6). In all cases, the model including a site class with *dN*/*dS*>1 fit significantly better than one without (*P*<10^−5^). The weakest evidence for positive selection came from the ficolins, which we were unable to verify as present in the venoms. In this toxin family, under both M2 and M8, only 1% of sites were estimated to be under selection, whereas >15% of sites were estimated to be under selection for all of the other data sets. We also ran three site-based selection analyses implemented in HyPhy [65, 68]. All three methods detected positively selected sites for all three SVMP domains (Table 7). We did not detect positively selected sites for 3FTxs with single-likelihood ancestor counting (SLAC), but did with fixed-effects likelihood (FEL) and random-effects likelihood (REL, Table 7). The evidence for positively selected sites was weakest for CTLs and ficolins; each only had evidence under one method.

**Table 5:** **Codeml selection analysis using the nearly neutral (M1) and the positive selection (M2) models**

Toxins	***n*** /model	M0: ***w***	M1: nearly neutral	-lnL	M2: positive selection	-lnL	***P***
SVMP-MP	18	1.15	*p*: (0.40, 0.60)	4,092.28	*p*: (0.31, 0.46, 0.23)	4,035.46	2.1×10^−25^
	GTR+G		*w*: (0.09, 1.00)		*w*: (0.08, 1.00, 3.85)		
SVMP-DIS	18	1.29	*p*: (0.45, 0.55)	1,388.59	*p*: (0.30, 0.54, 0.16)	1,357.60	3.5×10^−14^
	HKY+G		*w*: (0.09, 1.00)		*w*: (0.03, 1.00, 6.00)		
SVMP-ACR	18	1.39	*p*: (0.49, 0.51)	2,196.52	*p*: (0.28, 0.49, 0.23)	2,133.51	4.3×10^−28^
	HKY+G		*w*: (0.09, 1.00)		*w*: (0.00, 1.00, 6.03)		
3FTx	11	1.78	*p*: (0.33, 0.67)	1,300.12	*p*: (0.16, 0.44, 0.40)	1,269.82	6.9×10^−14^
	SYM+G		*w*: (0.07, 1.00)		*w*: (0.00, 1.00, 6.80)		
CTL	12	0.53	*p*: (0.45, 0.55)	3,921.91	*p*: (0.37, 0.44, 0.19)	3,902.58	4.0×10^−9^
	K80+I+G		*w*: (0.13, 1.00)		*w*: (0.14, 1.00, 3.26)		
Ficolin	7	0.37	*p*: (0.48, 0.52)	4,470.30	*p*: (0.47, 0.52, 0.01)	4,458.24	5.8×10^−6^
	GTR+G		*w*: (0.04, 1.00)		*w*: (0.04, 1.00, 51.68)		

*Abbreviations:* SVMP–snake venom metalloproteinase, MP–metalloproteinase domain, DIS–disintegrin domain, ACR–cysteine-rich domain, 3FTx–three-finger toxin, CTL–C-type lectin, *w*–ratio(s) of nonsynonyous to synonymous substitution rates, *p*–site-class frequencies, lnL–log likelihood, *n*–number of sequences.

**Table 6:** **Codeml selection analysis using the beta (M7) and the beta plus selection (M8) models**

Toxins	***n*** /model	M0: ***w***	M7:-lnL	M8: beta w/positive selection	-lnL	***P***
SVMP-MP	18	1.15	4,097.97	*p*: (0.75, 0.25)	4,036.79	2.7×10^−27^
	GTR+G			*w*: 3.78		
SVMP-DIS	18	1.29	1,389.94	*p*: (0.84, 0.16)	1,357.74	1.0×10^−14^
	HKY+G			*w*: 3.29		
SVMP-ACR	18	1.39	2,197.91	*p*: (0.77, 0.23)	2,133.66	1.2×10^−28^
	HKY+G			*w*: 5.97		
3FTx	11	1.78	1,300.92	*p*: (0.60, 0.40)	1,269.86	3.2×10^−14^
	SYM+G			*w*: 6.64		
CTL	12	0.53	3,913.45	*p*: (0.79, 0.21)	3,892.88	1.2×10^−9^
	K80+I+G			*w*: 2.57		
Ficolin	7	0.37	4,468.63	*p*: (0.99, 0.01)	4,456.99	8.8×10^−6^
	GTR+G			*w*: 43.40		

*Abbreviations:* SVMP–snake venom metalloproteinase, MP–metalloproteinase domain, DIS–disintegrin domain, ACR–cysteine-rich domain, 3FTx–three-finger toxin, CTL–C-type lectin, *w*–ratio of nonsynonyous to synonymous substitution rates, lnL–log likelihood, *n*–number of sequences.

**Table 7:** **HyPhy** [68] **selection analysis using the SLAC, FEL, and REL methods**

		Subst.	Total	SLAC codons	FEL codons	REL codons
Toxins	***n***	model	codons	***w***>1; ***P***<0 ***.***05	***w***>1; ***P***<0 ***.***05	***w***>1; ***B*** ***F***>100
SVMP-MP	18	REV	192	1	11	7
SVMP-DIS	17	HYK85	76	2	5	10
SVMP-ACR	17	HKY85	102	1	5	16
3FTx	11	REV	79	0	1	35
CTL	12	REV	137	0	2	0
Ficolin	7	REV	324	0	0	4

*Abbreviations:* SLAC–single-likelihood ancestor counting, FEL–fixed-effects likelihood, REL–random-effects likelihood, SVMP–snake venom metalloproteinase, MP–metalloproteinase domain, DIS–disintegrin domain, ACR–cysteine-rich domain, 3FTx–three-finger toxin, CTL–C-type lectin, *w*–ratio of nonsynonyous to synonymous substitution rates, *n*–number of sequences (duplicates removed), *BF*–Bayes factor.

The evidence for diversifying or positive selection on our putative toxin sequences was mixed. We found clear evidence for positive selection in the CRISPs (Figure 3), SVMPs, and at least some CTLs and 3FTxs (Figure 3 and Tables 5, 6, and 7). These putative toxins, perhaps not coincidentally, include most of those that were both at high levels in the venom-gland transcriptome and detected in the venom proteomes (Figures 1 and 2). The ficolins, which were not detected proteomically, showed weak evidence for a few sites being under selection (Tables 5, 6, and 7). Although this class of toxin is known from other colubrid snake species [57], they may serve nontoxic functions in ours, or perhaps only a subset of those detected play a toxic role, thereby diluting the signal for selection. Nonetheless, we can conclude that, like the venom components of viperids and elapids, the major types of toxins we identified in the venoms of *Hypsiglena* sp. and *B. irregularis* show strong signals for diversifying selection, which is consistent with their putatively toxic roles. Taxon-specific effects of *B. irregularis* (Guam) crude venom and purified Irditoxin, a heterodimeric 3FTx, have been demonstrated toward lizards and birds, whereas mammals (mice and humans) show minimal effects [38, 40]. Our results further suggest that the venoms of colubrids contribute significantly to their fitness, despite these venoms typically having little or no medically significant effects on humans.

### Divergent venom phenotypes

Our analyses revealed significant divergence between the two colubrids venoms, reflecting the dichotomy more typically observed between elapids and viperids. Optimal foraging theory predicts that ambush predators will be stocky and generally consume relatively large food items, whereas active predators will be slim and consume smaller prey [69]. A number of empirical and comparative studies have demonstrated these theoretical predictions in snakes [70–72]. Large relative prey masses have been reported for numerous stocky-bodied viperid species, such as *Crotalus oreganus* (with a mean of 0.40 [73]), *Bothrops moojeni* (with a mean of 1.08 [74]), and *Trimeresurus stejnegeri* (with a mean of 0.54 [75]). In contrast, small relative prey masses have been reported for slender, active foragers, particularly for arboreal rear-fanged colubrids such as *Psammodynastes pulverulentus* (with a mean of 0.13 [76]) and *Thelotornis capensis* (with a mean of 0.19 [77]). A mean relative prey mass of 0.24 for *Hypsiglena torquata sensu lato* and at least two cases of diurnal ambushing by these snakes have also been documented [21], traits comparable to what has been reported in viperids. The high abundance tissue-destroying toxins typical of most viperid venoms and found in *Hypsiglena* sp. could be critical for the rapid and efficient digestion of large prey items consumed by the ambush predators. On the other hand, the long, slender *B. irregularis* was reported to have a mean relative prey mass of 0.11 [78], lower than even other arboreal, active foraging, rear-fanged colubrids. Such small prey items are presumably simpler to digest given their higher surface area to volume ratio. Selection might have favored lethal neurotoxins to subdue prey, rather than abundant tissue-dissolving toxins, in species with active foraging ecologies.

## Conclusions

We presented the first comparative, high-throughput, transcriptomic analysis of the venom of two rear-fanged snakes with confirmatory peptide evidence from high-definition mass spectrometry. As previously seen for both elapids and viperids, venom expression was strongly biased towards toxin production in both *B. irregularis* and *Hypsiglena* sp., suggesting that venom plays an important function in the feeding ecology of these species. This inference of ecological importance was further supported by selection analyses, which showed strong evidence of diversifying selection for the major toxin classes. Although their venoms showed some diversity, these rear-fanged snakes expressed fewer toxin classes than their front-fanged counterparts. The extreme divergence observed between these two species in venom composition might be explained by their distinct foraging strategies. We also showed that, by taking advantage of high-sensitivity technologies, we can achieve complete qualitative venom characterization from single venom samples from single individual animals, despite low venom yields. Although initially requiring the sacrifice of an animal for venom glands, our transcriptome-directed proteomics approach can reduce the impact on native populations by allowing identification of toxins in the venom without long-term housing of animals and the need to combine the results of multiple venom extractions.

### Sequence accession numbers

The original, unmerged sequencing reads were submitted to the National Center for Biotechnology Information (NCBI) Sequence Read Archive under accession numbers SRR1292619 for *B. irregularis* and SRR1292610 for *Hypsiglena* sp. The assembled and annotated sequences were submitted to NCBI as Transcriptome Shotgun Assembly projects. The Transcriptome Shotgun Assembly projects have been deposited at DDBJ/EMBL/GenBank under the accessions GBSH00000000 for *B. irregularis* and GBSI00000000 for *Hypsiglena* sp.

## Methods

### Venom-gland tissues

An adult male *Hypsiglena* sp. specimen was collected in Cochise Co., AZ under permits from the Arizona Game and Fish Department to SPM (#SP677356). According to Mulcahy [20], two species occur in this county: *Hypsiglena* sp., an undescribed lineage, and *Hypsiglena jani texana*. Our specimen originated from near the town of Portal, AZ, further south than *Hypsiglena jani texana* is known to occur. However, because these two forms are distantly related, we also used the NADH dehydrogenase subunit 4 (ND4) sequences deposited in GenBank (EU363095 and EU363181) from Mulcahy [20] to compare to ND4 sequence (derived from the transcriptome described below) from our specimen. Our specimen was an identical match to *Hypsiglena* sp. and differed by more than 8% from the *Hypsiglena jani texana* sample, further confirming the identity of our specimen. Venom was extracted from the specimen using standard methods [79] (ketamine, 35 *μ*g/g; pilocarpine, 6 *μ*g/g). This specimen had a snout-vent length (SVL) of 335 mm and a tail length (TL) of 72 mm and weighed 28.9 g. An adult male *B. irregularis* specimen from Indonesia (exact locality unknown) was donated by United States Fish and Wildlife Service as an import confiscation. Venom was extracted from the specimen (SVL/TL = 1,315/290 mm, weight = 298 g) as above but dosing with ketamine at 20 *μ*g/g. Venom was centrifuged at 10k ×g for 5 minutes, and the supernatant was frozen at −80°C and lyophilized. Both animals were long-term captives.

Four days post-extraction, when mRNA levels were presumed maximized [80], both snakes were sacrificed by means of overdosing with isoflurane followed by decapitation. Both glands, which reside immediately below the lateral skin surfaces behind the eyes, were rapidly dissected from the snake, placed on clean Parafilm, and non-gland tissues (fat, connective tissue, muscle) were removed. Glands were then sliced into approximately 2 ×2 mm blocks with a sterile scalpel blade and placed in RNAlater. Treated glands were placed at 4°C for 2 hours and then stored at −80°C until used. All animal procedures were evaluated and approved by the University of Northern Colorado Institutional Animal Care and Use Committee (IACUC protocol 9204.1).

### RNA extraction

Venom-gland tissue was diced, placed in TRIzol (Invitrogen 15596-018), homogenized by mortar, and aspirated through a 20 gauge needle. The RNA was isolated from the lysate using a chloroform extraction in conjunction with Heavy Phase Lock Gel tubes (5 PRIME 2302810) and further purified by ethanol precipitation. Quality of the isolated RNA was assessed by Experion StdSens RNA Analysis Kit (Bio Rad). The mRNA was isolated using NEBNext Poly(A) mRNA Magnetic Isolation Module using 500 ng of total RNA for both *B. irregularis* and *Hypsiglena* sp.

### Sequencing

Library preparation was performed on the selected mRNA using NEBNext Ultra RNA Library Prep Kit and Multiplex Oligos for Illumina Sequencing (New England Biolabs). Incubation and PCR steps were carried out by Veriti Thermocycler (Applied Biosystems/Life). During and after the protocol, DNA was purified using Agencourt AMPure XP PCR Purification Beads. Size selection of adapter-ligated DNA was performed immediately prior to final library amplification. This step allowed us to optimize fragment-size distribution for sequencing. Size selection was performed according to NEBNext Ultra Protocol, Version 2.0. Final PCR amplification consisted of 12 cycles. Libraries were then quantified and assessed for quality using an Agilent 2100 Bioanalyzer. Samples were sequenced using the MiSeq Version 2 Reagent Kit on the Illumina MiSeq platform. Samples were prepared according to manufacturer’s protocol (revision B). Each sample was sequenced with a single kit with 151-nucleotide paired-end reads.

### Transcriptome assembly

The raw 151-nt paired-end reads passing the Illumina quality filter were merged if their 3’ ends overlapped as described previously [7, 11, 27]. This step also removed adapter sequences present because of fragment read-through. To eliminate reads corresponding to extremely high-abundance transcripts, we used the Extender program [27] with 1,000 merged reads as seeds to attempt to generate complete transcripts using only the merged reads. Extension of seeds required an overlap of 100 nucleotides, phred scores of at least 30 at each position in the extending read, and an exact match in the overlapping region. For *B. irregularis*, we performed a reference-based assembly against the 3,031 nontoxins previously annotated for *Crotalus horridus*[7] with NGen version 11.0 using both the merged and unmerged reads and a minimum match percentage of 85. Consensus sequences were retained if they had at least 5 × coverage over the entire coding sequence. Regions outside the coding sequence with less than 5 × coverage were removed. We performed the same type of assembly for *Hypsiglena* sp. except that we used the final set of nontoxins from *B. irregularis* as templates. Toxin sequences were clustered into groups with less <1% nucleotide divergence in their coding sequences, and duplicate nontoxin sequences were eliminated following alignment of the final transcripts with NGen. We used one representative from each toxin cluster and all of the unique nontoxins to filter the corresponding reads in a reference-based transcriptome assembly in NGen with a minimum match percentage of 98, using only the merged reads. The unfiltered reads were then used in a *de novo* transcriptome assembly in NGen with the default minimum match percentage of 93, retaining only contigs comprised of at least 100 reads.

To increase our chances of identifying all toxin sequences, we performed four additional *de novo* assemblies for each species. We ignored sequences without homology with known toxins for all four assemblies. Three assemblies were performed with NGen with a minimum match percentage of 98, using 1, 5, and 10 million reads. We opted for the high stringency for these assemblies to attempt to differentiate closely related paralogs and varied the number of reads because we found that some extremely high-abundance transcripts were difficult to assemble with too many reads, apparently because of low levels of unspliced transcripts. The fourth additional *de novo* assembly used the Extender program as above on 1,000 new random reads, and we only used this assembly to identify SVMPs, which were difficult for other methods to assemble.

After combining the results of all of the above assemblies and eliminating duplicates as described above, we performed one final round of read filtering of the merged reads, followed by a *de novo* assembly of the unfiltered reads as above, keeping only those contigs comprising ≥ 1,000 reads. We ignored sequences without homology with known toxins families and added any resulting unique toxins to our database. This step was included to ensure that we missed no toxin sequences with appreciable expression.

To screen for and eliminate potentially chimeric sequences in our toxin databases for both species, we first screened for evidence of recombination within each toxin family with GARD [81]. We used the general reversible model of sequence evolution and gamma-beta rates. If we found a signal for recombination resulting in significantly different tree topologies for different regions of the alignments based on KH tests, we performed a reference-based assembly with NGen version 11 with a minimum match percentage of 98 and the autotrim parameter set to false, using the toxin coding sequences as references and 10 million merged reads. Such high-stringency alignments facilitate the identification of chimeric sequences by producing either multimodal or extremely uneven coverage distributions, particularly in combination with our long, merged reads. Suspect sequences were confirmed to be chimeras of other sequences in our toxin database before removal.

Sequences were identified by means of blastx searches against the NCBI non-redundant (nr) protein database with a minimum E-value of 10^**−4**^ and retaining only 10 hits. *De novo* assembled transcripts were only retained and annotated if they had complete protein-coding sequences. Putative toxins were identified by searching their blastx match descriptions for toxin-related key words as described previously [7, 11, 27]. The final set of unique transcripts for each species was generated by combining the results from all assemblies and eliminating duplicates by means of an NGen assembly and a second, more stringent assembly in SeqMan Pro. Final transcript abundances were estimated by means of a reference-based transcriptome assembly with NGen with a minimum match percentage of 95, using only the coding sequences of transcripts. Signal peptides for toxins were identified by means of SignalP analyses [82]. Putative toxins were named with a toxin-class abbreviation, a number indicating cluster identity, and a lower-case letter indicating the particular member of a cluster.

### Proteomics

The Florida State University Translational Science Laboratory performed nanospray LC/MS^*E*^ using the Synapt G2 HD Mass Spectrometer with an integrated nanoAcquity UPLC (Waters Corp.) to analyze whole venom samples. The UPLC column was an Acquity UPLC BEH130 C18 with dimensions of 75 *μ*m × 250 mm and 1.7 *μ*m bead size. Digestion of the whole venom samples was performed using the Calbiochem ProteoExtract All-in-One Trypsin Digestion Kit (Merck, Darmstadt, Germany) according to the manufacturer’s instructions, using LC/MS grade solvents. Whole venom digests were adjusted to 3% acetonitrile in LC/MS grade water (J. T. Baker) with 0.1% formic acid and 24 fmol/ *μ*L yeast Alcohol Dehydrogenase 1 digest (Waters Corp.) as an internal standard. The second mobile phase (mobile phase B) was run using 0.1% formic acid in acetonitrile. The column was kept at 55°C. Sample load was optimized to reach a base peak signal intensity between 1.5–2.5 ×10^5^ (arbitrary units). The flow rate was 0.425 *μ*l/min and the gradient used was 7–35% mobile phase B/55 min and 35–50% mobile phase B/5 min. Glufibrinopeptide (785.8426 *m/z*, Waters Corp.) was used as the lock mass (external calibrant). The ionization mode was NanoESI Positive with a time of flight resolution setting of 20,000. Capillary voltage was 3.0 kV and cone voltage was 40 V. Nanoflow gas was set at 0 Bar, and the source temperature was 80°C. Acquistion range for the spectra was 50–2000 *m/z*, with collision energies of 4 V for MS and a 15–40 V ramp for MSE. Raw data were generated using MassLynx version 4.1 software (Waters Corp.) and data were processed in ProteinLynx Global SERVER version 3.0.

Proteins were identified using the PLGS Identity^*E*^ algorithm to search our transcriptome-derived databases. Databases were generated by taking all toxin and nontoxin mRNA sequences identified, translating them, and removing the signal peptide. These databases contained 3,215 sequences for *B. irregularis* and 2,815 sequences for *Hypsiglena sp*. Both databases had the internal standard, yeast Alcohol Dehydrogenase 1 (ADH1_YEAST, P00330), appended. A decoy database was generated by reversing the sequences of the original database. Decoys were concatenated with our databases and search simultaneously with the originals. Search parameters allowed for precursor and fragment mass tolerances to be set by the software. We allowed for one missed cleavage site, as well as post-translational modifications of cysteine carbamidomethylation and oxidation of methionine. We only accepted protein identifications if they had ≥3 matched peptides, ≥20% sequence coverage, and a higher protein score than the highest scoring decoy identified, resulting in a 0% False Positive Rate. Individual and group identifications were considered unique if they possessed at least one distinguishing peptide.

### Molecular evolutionary analyses

To test for evidence of unique evolutionary patterns among toxin orthologs, we generated null distributions of pairwise synonymous (*dS*) and nonsynonymous (*dN*) substitution rates and of the nonsynonymous to synonymous substitution rate ratio (*dN*/*dS*) for the nontoxins identified in the transcriptomes. We constructed amino-acid sequence databases for each species, excluding mitochondrially-encoded sequences, and conducted blastp searches of each sequence from each species against the database generated for the other with an E-value cutoff of 10^**−4**^. Putatively orthologous pairs were only retained if the two constituent sequences were each other’s best matches. The coding sequences of retained pairs were aligned using ClustalW [83]. Alignments with more than 24 gapped positions in the coding sequences were excluded from further consideration. For the remaining orthologous pairs, we estimated the pairwise synonymous (*dS*) and nonsynonymous (*dN*) substitution rates and the pairwise ratios of nonsynonymous to synonymous substitution rates (*dN*/*dS*) with codeml from PAML version 4.4 [66, 67]. A corresponding analysis was conducted for the putative toxin sequences to determine whether these pairs were outliers relative to the nontoxins. Differences in distributions were assessed by means of Wilcoxon rank sum tests.

To test for evidence of sites under selection for toxin classes for which we had three or more representatives between our two species, we first estimated maximum likelihood phylogenies with PAUP*, version 4.0b10 [84] and the iterative search strategy described by Rokyta et al. [85]. All alignments were constructed with ClustalW [83], and gapped positions were removed prior to any analyses. Evolutionary models were selected using MrModelTest version 2 with Akaike Information Criterion values. We used codeml from the PAML version 4.4. package [66, 67] to conduct a likelihood ratio test for positive selection. Specifically, we tested for the presence of a class of sites experiencing positive selection. Our null model was the nearly-neutral (M1) model in codeml, which allows for a class of sites evolving neutrally (*dN*/*dS*=1) and another class with its *dN*/*dS* estimated from the data but constrained to be <1. The alternative model, positive selection (M2), adds a third class with *dN*/*dS*>1. To test for positive selection, we compared negative twice the difference in log likelihoods between the models to a *χ*^**2**^ distribution with two degrees of freedom. We further confirmed our results by performing a similar test comparing models M7 (Beta) and M8 (Beta with positive selection), again using a *χ*^**2**^ distribution with two degrees of freedom [86]. To estimate an overall *dN*/*dS*, we used the M0 model, which fits a single ratio for all sites and branches, averaging *dN*/*dS* across sites and time. We further tested for evidence of positive selection acting on sites within alignments using HyPhy [68]. We used the same maximum-likelihood tree as for the codeml analysis and ran the SLAC, FEL, and REL methods [65]. In each case we used the substitution model most closely resembling the model selected with MrModelTest. For the SLAC and methods, we looked for site under selection with *P*<0.05, and for the REL methods, we looked for sites with Bayes factors ≥100.

## Abbreviations

3FTx: Three-finger toxin
AChE: Acetylcholinesterase
CDS: Coding sequence
CF: Coagulation factor
CTL: C-type lectin
CRISP: Cysteine-rich secretory protein
*dN*: Nonsynonymous substitution rate
*d**N*/*d**S*: Ratio of nonsynonymous to synonymous substitution rates
*dS*: Synonymous substitution rate
FEL: Fixed-effects likelhood
HYAL: Hyaluronidase
KUN: Kunitz-type protease inhibitor
NP: Natriuretic peptide
PDE: Phosphodiesterase
PLA_**2**_: **Phospholipase A**_**2**_
REL: Random-effects likelihood
SLAC: Single-likelihood ancestor counting
SVL: Snout-vent length
SVMP: Snake venom metalloproteinase
TL: Tail length
VEGF: Vascular endothelial growth factor
VESP: Vespryn (ohanin-like)
VF: Venom factor
WAP: Waprin.
